# Development and Immunogenic Evaluation of a Recombinant Vesicular Stomatitis Virus Expressing Nipah Virus F and G Glycoproteins

**DOI:** 10.3390/v17081070

**Published:** 2025-07-31

**Authors:** Huijuan Guo, Renqiang Liu, Dan Pan, Yijing Dang, Shuhuai Meng, Dan Shan, Xijun Wang, Jinying Ge, Zhigao Bu, Zhiyuan Wen

**Affiliations:** 1 State Key Laboratory of Animal Disease Control and Prevention, Harbin Veterinary Research Institute of Chinese Academy of Agricultural Sciences, Harbin 150069, China; 82101231355@caas.cn (H.G.); liurenqiang@caas.cn (R.L.); pdanvet@163.com (D.P.); 15214128050@163.com (Y.D.); 82101225721@caas.cn (S.M.); shandan@caas.cn (D.S.); wangxijun@caas.cn (X.W.); gejinying@caas.cn (J.G.); 2 Jiangsu Co-Innovation Center for Prevention and Control of Important Animal Infectious Diseases and Zoonoses, Yangzhou University, Yangzhou 225000, China

**Keywords:** Nipah virus, recombinant vesicular stomatitis virus, neutralizing antibodies

## Abstract

Nipah virus (NiV) is a highly pathogenic bat-borne zoonotic pathogen that poses a significant threat to human and animal health, with fatality rates exceeding 70% in some outbreaks. Despite its significant public health impact, there are currently no licensed vaccines or specific therapeutics available. Various virological tools—such as reverse genetics systems, replicon particles, VSV-based pseudoviruses, and recombinant Cedar virus chimeras—have been widely used to study the molecular mechanisms of NiV and to support vaccine development. Building upon these platforms, we developed a replication-competent recombinant vesicular stomatitis virus (rVSVΔG-eGFP-NiV_BD_ F/G) expressing NiV attachment (G) and fusion (F) glycoproteins. This recombinant virus serves as a valuable tool for investigating NiV entry mechanisms, cellular tropism, and immunogenicity. The virus was generated by replacing the VSV G protein with NiV F/G through reverse genetics, and protein incorporation was confirmed via immunofluorescence and electron microscopy. In vitro, the virus exhibited robust replication, characteristic cell tropism, and high viral titers in multiple cell lines. Neutralization assays showed that monoclonal antibodies HENV-26 and HENV-32 effectively neutralized the recombinant virus. Furthermore, immunization of golden hamsters with inactivated rVSVΔG-eGFP-NiV_BD_ F/G induced potent neutralizing antibody responses, demonstrating its robust immunogenicity. These findings highlight rVSVΔG-eGFP-NiV_BD_ F/G as an effective platform for NiV research and vaccine development.

## 1. Introduction

Nipah virus (NiV) is a highly pathogenic zoonotic virus designated as a priority pathogen by the World Health Organization due to its elevated fatality rate (40–75%) and potential for extensive outbreaks [1,2,3]. Since its emergence in Malaysia in 1998, NiV has caused recurrent outbreaks in South Asia and Southeast Asia, primarily through bat-to-human or intermediate host transmission [4,5,6]. The virus induces severe respiratory and neurological symptoms [7,8]. NiV belongs to the genus Henipavirus and possesses a single-stranded RNA genome encoding six proteins, among which the fusion (F) and attachment (G) glycoproteins play critical roles in viral entry into host cells [9,10,11]. The F protein mediates membrane fusion, whereas the G protein binds to host cell receptors, enabling viral attachment [12,13,14]. The synergistic function of the F and G proteins makes them key targets for the development of neutralizing antibodies and vaccines [15,16,17]. However, due to the virus’s high pathogenicity, related studies are strictly confined to biosafety level 4 (BSL-4) laboratories. In recent years, researchers have developed and applied various virological tools across platforms ranging from high biosafety level 4 (BSL-4) laboratories to systems operable under BSL-2 conditions, effectively advancing studies on Nipah virus (NiV) in molecular mechanisms, receptor interactions, and immunology. For example, researchers have constructed NiV ∆F replicon particles, which can be used under BSL-2 conditions and also hold potential as experimental vaccines [18]. Additionally, ZsGreen reporter strains of the Bangladesh (NiV_BD_) and Malaysia (NiV_MY_) isolates have been established in BSL-4 laboratories to facilitate viral replication and neutralization assays [19]. Other studies have reported recombinant Cedar virus chimeras displaying NiV F and G proteins on their surface, which can be used in lower biosafety environments for receptor binding and immunological research [20]. Earlier research also developed a VSV-ΔG-GFP pseudotyped system expressing NiV F and G proteins, used for studying viral entry and screening neutralizing antibodies [21]. Building on these foundations, this study employed a novel strategy to construct a replication-competent recombinant vesicular stomatitis virus (rVSVΔG-eGFP-NiV_BD_ F/G) expressing NiV attachment glycoprotein (G) and fusion glycoprotein (F). This system not only exhibits strong biological activity and operability but also provides a powerful tool for systematically elucidating NiV viral entry processes, host range, and induced immune responses, while establishing a new experimental platform for neutralizing antibody screening and vaccine evaluation.

The VSV platform possesses significant technical advantages, including well-characterized genetic manipulation, efficient viral replication capacity, and the ability to induce long-lasting immune responses. It has also been successfully applied in the development of vaccines against several highly pathogenic viruses, such as the Ebola virus, SARS-CoV-2, Marburg virus, Lassa fever virus, and Middle East respiratory syndrome coronavirus (MERS-CoV) [22,23,24,25,26]. In recent years, researchers have begun applying the VSV platform to the development of Nipah virus (NiV) vaccines. Current research primarily focuses on single glycoprotein expression strategies, where VSV recombinant viruses expressing either NiV G or F protein are constructed. Studies have demonstrated that a single-cycle recombinant VSV expressing NiV G or F protein can efficiently elicit protective immune responses in various animal models, including non-human primates, mice, and hamsters, whether administered alone or in combination [27,28,29,30,31]. These findings provide a solid foundation for the further development of NiV vaccines. Since the F and G proteins need to cooperate during the viral entry process, viruses expressing only a single glycoprotein cannot replicate independently. They require VSV-G assistance. Therefore, their application in viral entry studies remains limited. Additionally, the expression yield and stability may restrict the widespread application and commercialization of these vaccines. In this context, this study is the first to construct a recombinant VSV, rVSVΔG-eGFP-NiV_BD_ F/G, which simultaneously expresses both NiV Bangladesh strain F and G proteins. In this context, this study constructed for the first time a recombinant VSV virus, rVSVΔG-eGFP-NiV_BD_ F/G, which simultaneously expresses the F and G proteins of the Nipah virus Bangladesh strain. It aims to more accurately mimic the invasion mechanism of NiV. This virus has the potential for large-scale production and can serve as a powerful experimental tool for studies on host, cell, and tissue tropism, as well as for evaluating vaccine immunogenicity in both basic and applied research.

The in vitro experimental results demonstrated that the chimeric virus could effectively mimic the receptor-binding and membrane fusion functions of Nipah virus, and exhibited a cell tropism highly consistent with that of the wild-type virus, validating its reliability and practicality as a surrogate model.

To evaluate the application of the recombinant virus rVSVΔG-eGFP-NiV_BD_ F/G, we first tested the neutralizing capacity of the monoclonal antibodies HENV-26 and HENV-32. The results showed that both antibodies effectively neutralized the recombinant virus, which was consistent with previous findings [32]. The control virus rVSV-eGFP remained unneutralized, confirming assay specificity and establishing this platform as a reliable tool for NiV antibody screening. Next, we assessed the immunogenicity of the rLa-NiV F and rLa-NiV G vaccines. Using the recombinant virus, we quantified in vitro neutralizing antibody titers in immunized animal sera. Vaccinated animals developed potent antibodies targeting NiV F/G proteins, and the virus effectively detected these vaccine-induced immune responses, demonstrating its applicability for preclinical vaccine evaluation.

Although the VSV vector is generally considered a safe and suitable platform for vaccine development, its pathogenicity may change significantly after incorporating surface glycoproteins from different viruses. For example, previous studies have shown that VSV-MARV/GP and VSV-EBOV can cause severe clinical symptoms and even death in hamsters, suggesting that the safety of the VSV vector is influenced not only by its backbone but also by the biological characteristics of the inserted antigen and the sensitivity of the host species [33,34]. Studies have reported that simultaneous intranasal or intracranial inoculation with rVSVΔG-eGFP-NiV_BD_ F and rVSVΔG-eGFP-NiV_BD_ G can cause death in mice [35]. Notably, the application of the VSV vector in vaccine research has been widely validated, and both the VSV G protein and the NiV F and G proteins possess certain neurotropic properties. Therefore, intranasal or intracranial inoculation may induce neurological infections in mice.

In this study, we also observed that the recombinant virus rVSVΔG-eGFP-NiV_BD_ F/G replicated efficiently in BHK cells during the virus rescue process, further indicating that it may possess strong replication capability and pose potential pathogenic risks in the hamster animal model. To minimize risks and ensure vaccine safety, we employed immunologically inactivated recombinant viral particles for vaccine evaluation in subsequent experiments. Although inactivation may attenuate some of the inherent immunological advantages of the VSV vector, considering the high viral titers and yields of this recombinant virus and its efficient expression of NiV F and G proteins on the viral surface, our results demonstrated that the inactivated rVSVΔG-eGFP-NiV_BD_ F/G still elicited strong immunogenicity in this animal model, providing robust experimental evidence for the further development of Nipah virus vaccines.

This study established rVSVΔG-eGFP-NiV_BD_ F/G as a versatile and sensitive tool for investigating the invasion mechanism of Nipah virus, screening therapeutic antibodies, and potentially accelerating vaccine development.

## 2. Materials and Methods

### 2.1. Cells, Viruses, and Antibodies

The following cell lines were used in this study: African green monkey kidney cells (Vero, sourced from ATCC, Manassas, VA, USA, catalogue number CCL-81), baby Syrian hamster kidney cells (BHK-21, sourced from ATCC, catalogue number CCL-10), rabbit kidney 13 cells (RK-13, sourced from ATCC, catalogue number CCL-37), human embryonic kidney cells (HEK-293, sourced from ATCC, catalogue number CRL-1573), human alveolar basal epithelial cells (A549, sourced from ATCC, catalogue number CCL-185), Crandell–Rees feline kidney cells (CRFK, sourced from ATCC, catalogue number CCL-94), equine embryo kidney cells (Eqek, provided by Professor Du Cheng from the Chinese Academy of Agricultural Sciences), Madin–Darby bovine kidney cells (MDBK, sourced from ATCC, catalogue number CCL-22), porcine kidney 15 cells (PK-15, sourced from ATCC, catalogue number CCL-33), Madin–Darby canine kidney cells (MDCK, sourced from ATCC, catalogue number CCL-34), Chinese hamster ovary cells (CHO-K1, sourced from ATCC, catalogue number CCL-61), and swine testicular cells (ST, sourced from ATCC, catalogue number CRL-1746). These cells were cultured in Dulbecco’s modified Eagle’s medium (DMEM, Gibco, Grand Island, NY, USA, catalogue number 11995073) supplemented with 10% fetal bovine serum (FBS, ExCell, catalogue number 10099141), 1% L-glutamine, and 1% penicillin-streptomycin. Cells were incubated at 37 °C with 5% CO_2_. Porcine alveolar macrophages (PAMs) were cultured in Roswell Park Memorial Institute-1640 medium (RPMI-1640, Sigma, St. Louis, MO, USA) supplemented with 10% FBS, 1% L-glutamine, and 1% penicillin-streptomycin and incubated at 37 °C with 5% CO_2_. The recombinant virus rVSV-eGFP was constructed and stored in our laboratory. Monoclonal antibodies used in this study included: anti-HeV/NiV fusion glycoprotein F0 (Clone 5B3; AntibodySystem, Paris, France), and anti-HeV/NiV glycoprotein G (Clones HENV-26 and HENV-32; AntibodySystem, France). Our laboratory prepared mouse anti-NiV F protein serum and mouse anti-NiV G protein serum for indirect immunoassay and immunoelectron microscopy experiments. Pig serum from pigs immunized with the recombinant viruses rLa-NiV F and rLa-NiV G were also prepared and stored in our laboratory [36].

### 2.2. Experimental Animals

Five-week-old golden hamsters were purchased from Beijing Vital River Laboratory Animal Technology Co., Ltd (Keji Park, Beijing, China). All the animals were housed in isolation at the Animal Experimental Center of Harbin Veterinary Research Institute, Chinese Academy of Agricultural Sciences, and were provided with sterile feed and water. The experimental protocol and facility setup were approved by the Animal Ethics Committee of the Harbin Veterinary Research Institute, Chinese Academy of Agricultural Sciences (Approval No.: 240408-01-GR).

### 2.3. Rescue of rVSVΔG-eGFP-NiV_BD_ F/G

The VSV *G* gene was excised from the full-length cDNA plasmid pCI-Rz-VSV-eGFP-FL, which was preserved in our laboratory, through *NheI* digestion [25]. The linearized vector fragment (~15 kb) was purified through agarose gel electrophoresis. The plasmids pCAGGS-NiV_BD_ F and pCAGGS-NiV_BD_ G were synthesized from Jilin Kumei Biotechnology Co., Ltd (Beihu Science and Technology Development Zone, Changchun, China), and they were used as templates for PCR amplification of the NiV F and NiV G genes of the Bangladesh strain. After electrophoretic separation and recovery, the NiV F/G fragments were ligated into the linearized VSV vector using homologous recombination, ensuring that each gene was expressed as an independent open reading frame (ORF) rather than being fused together. The recombinant constructs were transformed into DH5α competent cells, and positive clones were screened using colony PCR. Plasmid integrity was confirmed through *Nhe*I digestion and sequencing. The final construct was designated rVSVΔG-eGFP-NiV_BD_ F/G.

To enable efficient virus rescue, ORFs of the *N*, *P*, *L*, and *G* genes were PCR-amplified from pCI-Rz-VSV-*eGFP*-FL and subcloned and inserted into the pCAGGS vector. The resulting helper plasmids, pVSV-N, pVSV-P, pVSV-*L*, and pVSV-*G*, were critical for providing essential viral proteins during rescue experiments.

BHK cells were plated in 6-well plates (5 × 10^4^ cells/well). Upon reaching ~80% confluency, the cells were transfected with a mixture containing 2 μg of pVSVΔG-*eGFP-NiV_BD_ F/G*, 2 μg of pVSV-*N*, 2 μg of pVSV-*P*, 1 μg of pVSV-*L*, and 1 μg of pVSV-*G* via X-tremeGENE HP DNA Transfection Reagent (Roche, 2:1 ratio). After 6–8 h, the medium was replaced with DMEM containing 10% FBS. Fluorescence microscopy (Zeiss) at 36 h post-transfection revealed *eGFP* expression in >90% of cells. The supernatant was then collected, centrifuged (1000× *g*, 5 min) to remove debris, and passaged onto fresh BHK cells. Once cytopathic effects (CPEs) affected >90% of the cells, the recombinant virus *rVSVΔG-eGFP-NiV_BD_ F/G* was harvested, aliquoted, and stored at −80 °C.

### 2.4. Western Blot

Vero cells were seeded in 6-well plates at a density of 2 × 10^5^ cells per well. After overnight incubation, the cells were infected with rVSVΔG-eGFP-NiV_BD_ F/G or rVSV-eGFP at a multiplicity of infection (MOI) of 0.01. At 36 h post-infection, the culture supernatant was collected and clarified by low-speed centrifugation (2000× *g*, 10 min, 4 °C) to remove cellular debris. The clarified supernatant was then subjected to ultracentrifugation through a 20% (*w*/*v*) sucrose cushion at 80,000× *g* for 4 h at 4 °C to pellet the viral particles. The virus pellet was resuspended in cold PBS and lysed on ice for 30 min using RIPA lysis buffer (Beyotime, Shanghai, China) containing protease inhibitors (Beyotime).

The lysates of purified virions were centrifuged at 12,000× *g* for 10 min at 4 °C to remove insoluble material. The supernatant was collected, mixed with 5× protein loading buffer, boiled for 5 min, and loaded onto a 12% SDS-PAGE gel. Electrophoresis was performed at 120 V for 1 h. After electrophoresis, proteins were transferred to a PVDF membrane (Millipore, Billerica, MA, USA) using a semi-dry transfer system at 15 V for 1 h.

The membrane was blocked with PBS-T (0.1% Tween-20) containing 5% nonfat milk for 2 h at room temperature and then washed three times with PBS-T, each wash lasting 10 min. The membrane was incubated overnight at 4 °C with mouse monoclonal antibodies against Nipah virus F and G proteins (1:1000). After washing, the membrane was incubated with IRDye 680RD-labeled goat anti-mouse IgG (1:5000) for 1 h at room temperature. Protein bands were visualized using the Odyssey infrared imaging system (LI-COR, Lincoln, NE, USA).

### 2.5. Immunofluorescence Assay

BHK cells were seeded in 6-well plates at a density of 2 × 10^5^ cells/well. The cells were infected with rVSVΔG-eGFP-NiV_BD_ F/G or rVSV-eGFP at an MOI of 0.01. After 1 h of infection at 37 °C, the cells were washed three times with PBS and then cultured in a fresh medium until cytopathic effects (CPEs) reached 80–90%. The cells were fixed with 4% paraformaldehyde at room temperature for 15 min and washed three times with PBS. Fixed cells were permeabilized with PBS containing 0.1% Triton X-100 at room temperature for 15 min and washed three times with PBS. The cells were blocked with PBS containing 1% bovine serum albumin (BSA) for 1 h at room temperature to reduce non-specific binding. The cells were incubated with mouse anti-NiV F protein serum (1:500) and mouse anti-NiV G protein serum (1:500) as primary antibodies for 1 h at room temperature. After three washes with PBS, the cells were incubated with Dylight 549-conjugated goat anti-mouse IgG (1:1000, Abcam, Cambridge, UK) for 1 h at room temperature. The cells were washed three times with PBS, and fluorescent expression was observed using an inverted fluorescence microscope (Carl Zeiss AG, Oberkochen, Germany). Images were captured and analyzed via ZEN software (Version 3.8, Carl Zeiss AG, Oberkochen, Germany).

### 2.6. Microscopic Observation

Purified rVSVΔG-eGFP-NiV_BD_ F/G and rVSV-eGFP viral particles were subjected to negative staining and analyzed using transmission electron microscopy (TEM, Hitachi High-Technologies Corporation, Tokyo, Japan) operated at an acceleration voltage of 80 kV. For immunogold labeling, recombinant virus particles were incubated with mouse anti-NiV F and G protein sera, followed by incubation with a 1:100 dilution of anti-mouse IgG (whole molecule)—Gold, antibody produced in goat (Sigma, G7652). Gold particles bound to the viral surface confirmed the presence of NiV glycoproteins.

### 2.7. In Vitro Growth Kinetics

Vero cells were seeded in 24-well plates at 1 × 10^5^ cells/well. The cells were infected with rVSVΔG-eGFP-NiV_BD_ F/G or VSV-eGFP at an MOI of 0.01 in serum-free DMEM for 1 h. After infection, the inoculum was removed, the cells were washed 3 times with PBS, and fresh DMEM supplemented with 2% FBS was added. The supernatants were collected every 12 h from 0 to 60 h post-infection, centrifuged at 1000× *g* for 5 min, and stored at −80 °C. Viral titers were determined via a TCID_50_ assay. Serial 10-fold dilutions of supernatants were prepared, and 100 µL of each dilution was added to Vero cells in 96-well plates (8 replicates per dilution). The plates were incubated for 2 to 3 days, after which the cytopathic effects (CPEs) were recorded. TCID_50_ values were calculated via the Reed–Muench method and expressed as log10 TCID_50_/mL.

### 2.8. Immunization of Golden Hamsters

The recombinant virus rVSVΔG-eGFP-NiV_BD_ F/G was inactivated with β-propiolactone at a ratio of 1:3000 (*v*/*v*) overnight at 4 °C, followed by treatment at 37 °C for 2 h the next day. The inactivated virus was purified by centrifugation at 100,000× *g* for 2 h at 4 °C using a layered discontinuous sucrose density gradient (20% to 60%). The virus band was collected, and another centrifugation at 100,000× *g* for 2 h at 4 °C was performed to remove sucrose. The virus was then dissolved in PBS. Protein concentration was determined using the BCA protein assay kit (Beyotime) according to the manufacturer’s protocol.

To confirm complete inactivation of rVSVΔG-eGFP-NiV_BD_ F/G by β-propiolactone, a virus inactivation validation assay was performed. Following inactivation and purification, 100 µL of the treated virus preparation was inoculated onto BHK cell monolayers and cultured at 37 °C with 5% CO_2_ for 3 days. Cells were observed daily for cytopathic effects (CPE) and eGFP expression under a fluorescence microscope. After 3 days, supernatants were collected and passaged onto fresh BHK cells for another 7-day incubation under the same conditions. No CPE or fluorescence signal was detected throughout two passages, indicating that the inactivation was complete and no residual infectious virus remained. Meanwhile, no bacterial contamination or other obvious cytopathic effects were observed in the cells.

Ten five-week-old female golden hamsters (strains: LVG SYR) were randomly divided into two groups. The inactivated virus (30 μg/animal) was mixed with Montanide GEL02 adjuvant (Seppic, Paris, France) at a 9:1 (*v*/*v*) ratio and administered via intramuscular injection in a total volume of 100 μL. Booster immunizations were performed 3 weeks later using the same dose and via the same route. The control group was injected with an equivalent volume of PBS mixed with the adjuvant.

Blood samples were collected via the retro-orbital route at 0, 2, 3, 4, 5 and 6 weeks post-immunization. The serum was separated via centrifugation at 2000× *g* for 10 min, inactivated at 56 °C for 30 min, aliquoted, and stored at −80 °C to avoid repeated freeze—thaw cycles.

### 2.9. Neutralization Assay

The samples to be tested were serially diluted two-fold in a 96-well flat-bottom tissue culture plate, serial dilutions starting at 1:2, with 25 μL of each dilution per well. Each serum dilution was mixed with an equal volume of rVSVΔG-eGFP-NiV_BD_ F/G virus solution containing 100 TCID_50_ in DMEM. The serum–virus mixture was incubated for 1 h. Vero cells were seeded in 96-well plates at a density of 1 × 10^4^ cells per well and cultured until they reached 80–90% confluency. Then, 50 μL of the serum–virus mixture was added to the pre-seeded Vero cells and incubated at 37 °C with 5% CO_2_ for 1 h. Subsequently, 100 μL of fresh DMEM supplemented with 2% FBS was added to each well, and the cells were further incubated at 37 °C. At 36 h post-infection, cytopathic effects (CPEs) were observed using an inverted fluorescence microscope (Carl Zeiss AG, Oberkochen, Germany). Viral neutralization titers (VNTs) were calculated using the Reed–Muench method on the basis of the highest serum dilution that inhibited virus-induced cytopathic effects (CPEs).

### 2.10. Statistical Analysis

The *p*-values for statistical analysis were determined using the One-way ANOVA function in GraphPad Prism 9.0, followed by Bonferroni’s multiple comparison test. *p* value < 0.05 was considered statistically significant.

## 3. Results

### 3.1. Rescue of the rVSVΔG-eGFP-NiV_BD_ F/G

**Figure 1:**
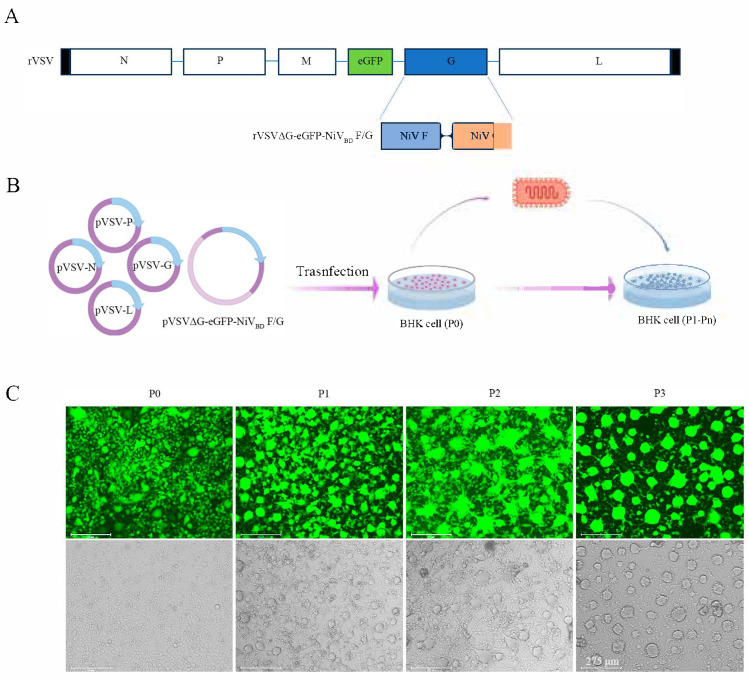
Generation and characterization of rVSVΔG-eGFP-NiV_BD_ F/G: (**A**) Schematic representation of the genomic organization of VSV-eGFP and rVSVΔG-eGFP-NiV_BD_ F/G. The viral genome (3′ to 5′ orientation) contains the *N* (nucleocapsid), *P* (phosphoprotein), *M* (matrix), *G* (glycoprotein), and *L* (RNA-dependent RNA polymerase) genes. The enhanced green fluorescent protein (eGFP) was inserted downstream of the M gene as a reporter for infection. The VSV glycoprotein (G) was replaced with the NiV F and G gene segments to generate rVSVΔG-eGFP-NiV_BD_ F/G. (**B**) Schematic overview of the generation process for rVSVΔG-eGFP-NiV_BD_ F/G. The pVSVΔG-eGFP-NiV_BD_ F/G plasmid, along with helper plasmids, was transfected into BHK cells to produce the primary virus stock (P0). The supernatant from these cells was subsequently used to infect fresh BHK cells for serial passages. (**C**) Serial passage of rVSVΔG-eGFP-NiV_BD_ F/G. When green fluorescence was observed in more than 90% of the cells, both the supernatant and virus-infected cells were harvested for further passages. Scale bar: 275 μm.

To engineer a VSV-based vector expressing NiV F and G glycoproteins, the VSV *G* gene in the pVSVΔG-eGFP-NiV_BD_ F/G plasmid was replaced with the NiV F and G genes (Figure 1A). Leveraging the VSV reverse genetics platform, BHK cells were co-transfected with the modified plasmid and helper plasmids (pVSV-N, pVSV-P, pVSV-L, pVSV-G) at a 2:2:2:1:1 ratio (Figure 1B). At 36 h post-transfection, >90% of the cells displayed green fluorescence, confirming efficient transfection. The resulting supernatant (P0) was passaged onto naïve BHK cells, resulting in the formation of multinucleated giant cells—a hallmark of NiV-induced cytopathic effects characterized by cell fusion and syncytia formation (Figure 1C). Viral genome sequencing verified the precise integration of the NiV *F* and *G* genes without unintended mutations. We have submitted the data reported in this paper to the GenBase database at the National Genomics Data Center (NGDC), with the accession number C_AA110798. The data are publicly accessible at https://ngdc.cncb.ac.cn/genbase.

### 3.2. Expression and Localization of NiV F and G Proteins

**Figure 2:**
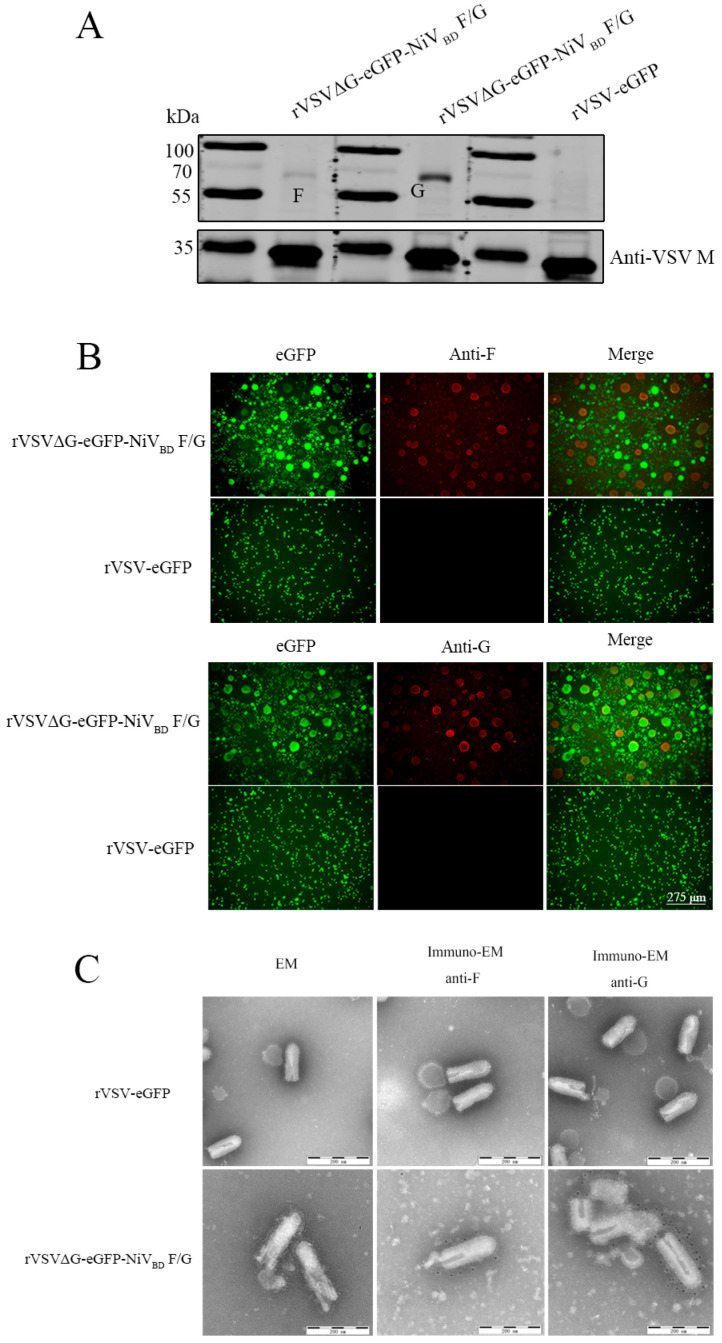
Characterization of rVSVΔG-eGFP-NiV_BD_ F/G: (**A**) Western blot analysis of purified rVSV-eGFP and rVSVΔG-eGFP-NiV_BD_ F/G. The blot was probed with commercial monoclonal antibodies against NiV G (HENV-26) and NiV F (5B3) to confirm the presence of the respective proteins. (**B**) Immunofluorescence analysis of NiV F and G protein expression in BHK cells infected with rVSVΔG-eGFP-NiV_BD_ F/G or rVSV-eGFP. The cells were stained with monoclonal antibodies against NiV G (HENV-26) and NiV F (5B3), followed by an Alexa Fluor 594-conjugated secondary antibody. Scale bar = 275 μm. (**C**) Transmission electron microscopy (TEM) and immunoelectron microscopy (IEM) of recombinant virus particles. Concentrated rVSVΔG-eGFP-NiV_BD_ F/G and rVSV-eGFP were negatively stained with phosphotungstic acid and probed with monoclonal antibodies against NiV G (HENV-26) and NiV F (5B3), followed by gold-conjugated secondary antibodies. Scale bar = 200 nm.

To verify the expression of viral proteins in the recombinant virus, we performed Western blot and immunofluorescence assays. Western blot analysis revealed specific bands of approximately 70 kDa in the purified rVSVΔG-eGFP-NiV_BD_ F/G samples, corresponding to the NiV F/G glycoproteins, whereas no such bands were detected in the rVSV-eGFP control group. In addition, the VSV matrix protein (M protein) was used as an internal control and loading reference to confirm the integrity of the viral particles in the samples (Figure 2A). Immunofluorescence further confirmed robust expression of NiV F and G proteins in BHK cells infected with rVSVΔG-eGFP-NiV_BD_ F/G, as indicated by strong red fluorescence signals with evident colocalization. No fluorescence signal was observed in the control group (Figure 2B).

To assess protein localization, immunoelectron microscopy (IEM) demonstrated gold-labelled anti-NiV F or anti-NiV G antibodies specifically bound to the envelope of rVSVΔG-eGFP-NiV_BD_ F/G virions, with no labelling observed on rVSV-eGFP particles (Figure 2C). TEM further showed that recombinant virions maintained the bullet-shaped morphology characteristic of wild-type vesicular stomatitis virus (VSV). Notably, Figure 2C shows that the particle size of rVSVΔG-eGFP-NiV_BD_ F/G virions is significantly larger than that of rVSV-eGFP virions. This difference may be related to the insertion of the NiV F/G genes, which increased the viral genome by approximately 2000 nucleotides and could potentially lead to the elongation of the viral particles. These results conclusively demonstrate the functional incorporation of NiV glycoproteins into the recombinant virus, establishing its utility for studying NiV entry and pathogenesis.

### 3.3. Characterization of rVSVΔG-eGFP- NiV_BD_ F/G

**Figure 3:**
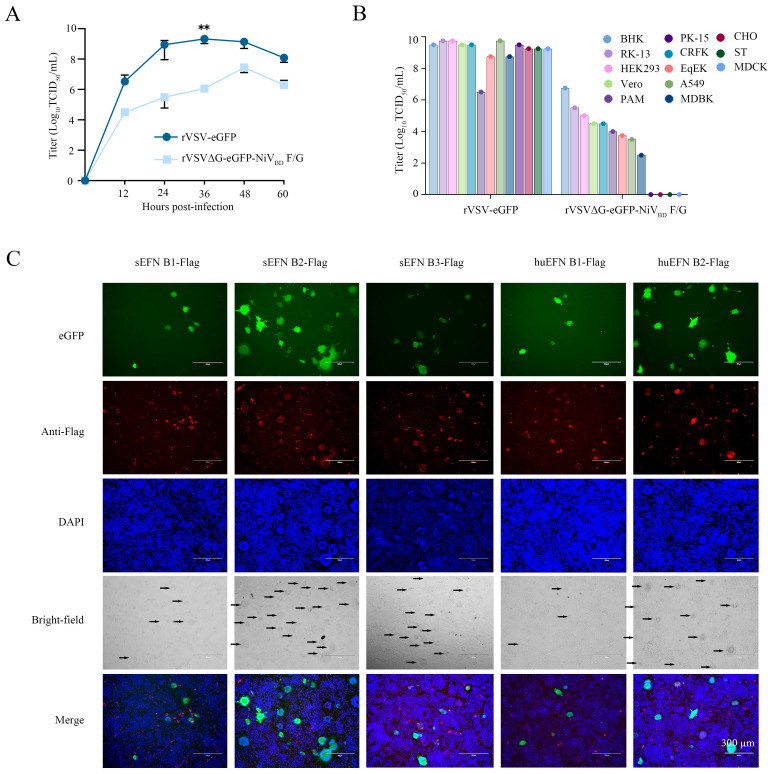
Characterization of the recombinant rVSVΔG-eGFP-NiV_BD_ F/G virus: (**A**) Growth kinetics of rVSVΔG-eGFP-NiV_BD_ F/G and rVSV-eGFP in Vero cells. Viral titers were measured at 12, 24, 36, 48, and 60 h post-infection using the TCID_50_ assay. **, *p* < 0.01 (**B**) cell tropism of rVSVΔG-eGFP-NiV_BD_ F/G. The infectivity of the recombinant virus was assessed in various cell lines, including BHK, EqEK, MDCK, RK-13, A549, HEK293, MDBK, Vero, PK-15, CRFK, CHO, PAM, and ST cells. Viral titers were determined 48 h post-infection. (**C**) Ephrin receptor dependency of rVSVΔG-eGFP-NiV_BD_ F/G. MDCK cells expressing sEFN B1, B2, B3, and huEFN B1, B2 were infected with rVSVΔG-eGFP-NiV_BD_ F/G. Immunofluorescence staining was performed with an anti-Flag antibody (red) to detect Ephrin receptor expression, and nuclei were counterstained with DAPI (blue). Scale bar: 300 µm.

To determine the impact of the insertion of the NiV F and G genes on the replication ability of the recombinant virus, we measured the growth kinetics of rVSVΔG-eGFP-NiV_BD_ F/G and rVSV-eGFP in infected Vero cells. The results showed that the recombinant virus rVSVΔG-eGFP-NiV_BD_ F/G reached a peak viral titer of 10^7.25^ TCID_50_/mL at 48 h, although it had slightly lower titers and slower growth kinetics compared to rVSV-eGFP (*p* < 0.01) (Figure 3A). This indicated that replacing the VSV G protein with the NiV F and G proteins significantly affects the ability of the virus to replicate.

To assess the infectivity of the recombinant virus, we measured the viral titers of rVSV-eGFP and rVSVΔG-eGFP-NiV_BD_ F/G in various cell lines (Figure 3B). The results showed that rVSV-eGFP exhibited high infectivity in all the tested cell lines, retaining the broad host tropism of the parental VSV. In contrast, the infectivity of rVSVΔG-eGFP-NiV_BD_ F/G was significantly cell line-dependent, with high infectivity observed in BHK, RK-13, HEK293, A549, Vero, CRFK, EqEK, and MDBK cells, but no infection detected in PK-15, MDCK, CHO, and ST cells. These findings suggest that the host tropism of the recombinant virus has been altered, and that rVSVΔG-eGFP-NiV_BD_ F/G possesses a broad potential host range.

**Figure A1:**
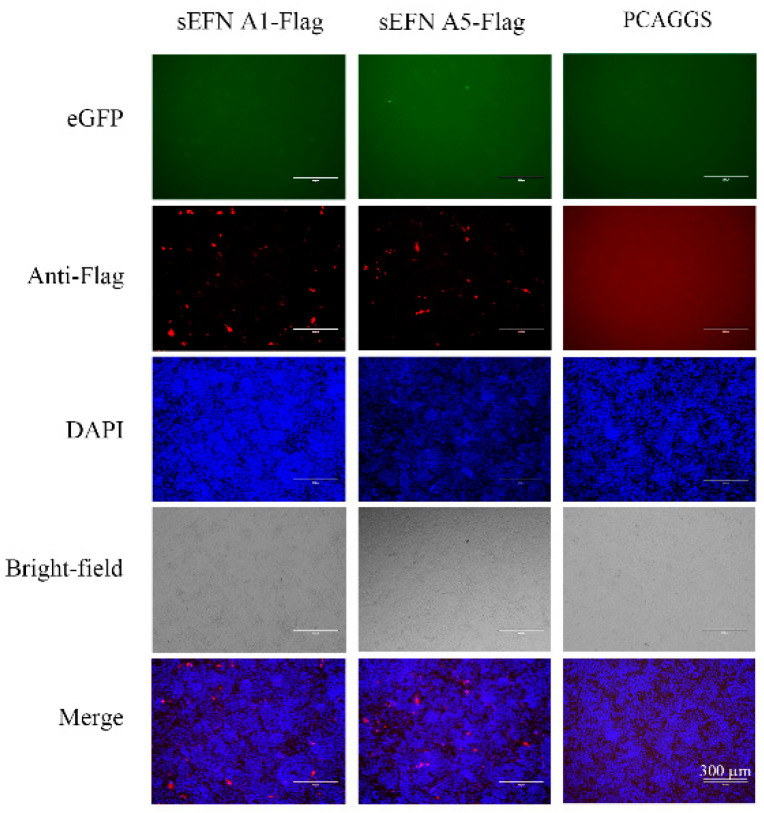
Ephrin receptor dependency of rVSVΔG-eGFP-NiV_BD_ F/G: MDCK cells expressing sEFN A1, A5, and the empty vector PCAGGS were infected with rVSVΔG-eGFP-NiV_BD_ F/G. Immunofluorescence staining was performed with an anti-Flag antibody (red) to detect Ephrin receptor expression, and nuclei were counterstained with DAPI (blue). Scale bar: 300 µm.

On the basis of the above results, we selected MDCK cells as target cells because they do not support NiV F and G protein-mediated viral infection. We cloned members of the EFN receptor family from Vero cells and 293T cells, including monkey-derived EFN receptors: EFN A1 (GenBank: XM 007976898.2), EFN A5 (GenBank: XM 008014091.2), EFN B1 (GenBank: XM 007991957.2), EFN B2 (GenBank: XM 007960854.2), and EFN B3 (GenBank: XM 008010196.2), as well as human-derived EFN receptors: EFN B1 (GenBank: NM_004429) and EFN B2 (GenBank: NM_001372056). By transfecting these EFN receptor family members into cells, we investigated their roles in viral infection. The results showed that cells transfected with sEFN B1, sEFN B2, sEFN B3, huEFN B1, or huEFN B2 can be infected by the recombinant virus rVSVΔG-eGFP-NiV_BD_ F/G, as indicated by the expression of enhanced green fluorescent protein (eGFP). Meanwhile, cells expressing sEFN A1 or A5 showed no fluorescence or cytopathic effects (Figure A1). Among these, sEFN B2, sEFN B3, and huEFN B2 cells also exhibited significant syncytium formation. In contrast, cells expressing sEFN B1 or huEFN B1 showed lower levels of fusion and infection efficiency. Although their infection effects were less pronounced than those of B2 and B3, it still suggests that B1 might play an auxiliary role in NiV-mediated viral entry, albeit weak (Figure 3C). These findings suggest that while the potential role of B1 in NiV entry is relatively weak, it should not be overlooked and still holds some research value.

The results of this study demonstrate that rVSVΔG-eGFP-NiV_BD_ F/G closely mimics the biological characteristics of NiV in terms of replication kinetics, host tropism, and receptor binding properties, providing an important tool for studying the mechanisms of NiV entry and receptor distribution.

### 3.4. rVSVΔG-eGFP-NiV_BD_ F/G as a Versatile Tool for Neutralizing Antibody Detection and Vaccine Efficacy Studies

**Figure 4:**
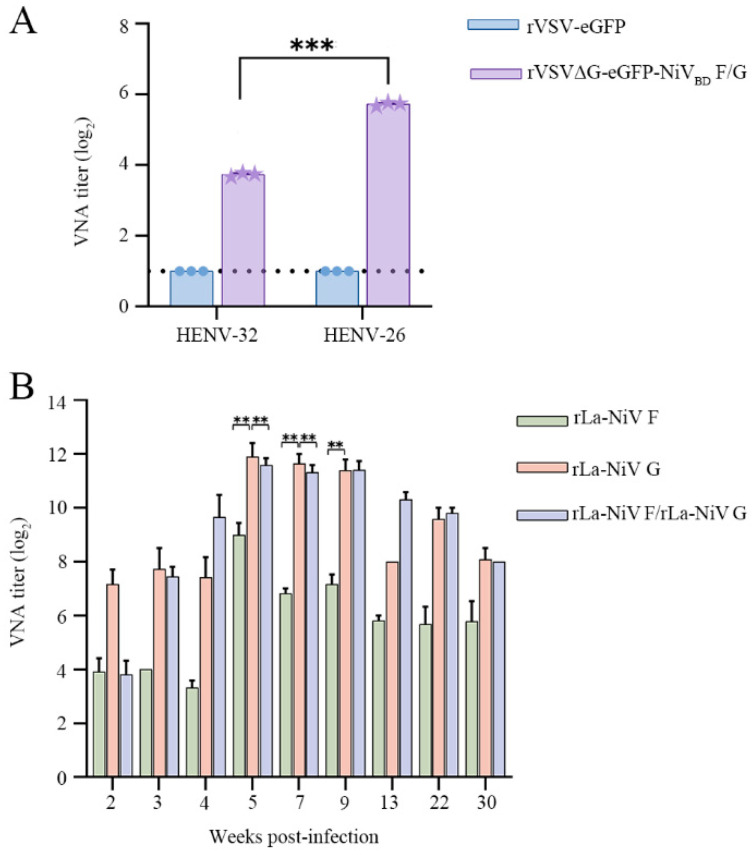
Application of recombinant rVSVΔG-eGFP-NiV_BD_ F/G in neutralization assays: (**A**) Neutralization potency of the monoclonal antibodies HENV-26 and HENV-32 against rVSVΔG-eGFP-NiV_BD_ F/G. The neutralizing antibody (VNA) titers of the HENV-26 and HENV-32 were determined against rVSVΔG-eGFP-NiV_BD_ F/G and the control virus rVSV-eGFP. The data are presented as log2 VNA titers. ***, *p* < 0.001. (**B**) Neutralizing antibody responses in pigs immunized with rLa-NiV F, rLa-NiV G, or rLa-NiV F/rLa-NiV G. Neutralizing antibody titers were measured at 1, 2, 3, 4, 5, 7, 9, 13, 22, and 30 weeks post-immunization. The data are presented as log2 VNA titers. The error bars represent the standard deviation of the mean (*n* = 4). **, *p* < 0.01.

This study evaluated the potential applications of the recombinant virus rVSVΔG-eGFP-NiV_BD_ F/G in neutralizing antibody detection and receptor function research. The research reports indicate that the monoclonal antibodies HENV-26 and HENV-32 can effectively neutralize Nipah virus (NiV) and prevent viral entry. To validate the potential of the recombinant virus in neutralization assays, we established a neutralization system based on HENV-26 and HENV-32. The results showed that both monoclonal antibodies effectively neutralized the recombinant virus, with HENV-26 showing significantly higher neutralizing activity than HENV-32 (*p* < 0.001), consistent with previous studies on wild-type viruses (Figure 4A). The control virus rVSV-eGFP showed no neutralizing ability, confirming the specificity of the assay. These results demonstrate that the recombinant virus replicates the neutralization response characteristics of wild-type NiV, providing a safe and efficient experimental tool for neutralizing antibody screening while avoiding the high risks associated with handling live viruses.

In this study, we further evaluated the neutralizing antibody levels against Nipah virus (NiV) using laboratory-prepared immune porcine sera derived from pigs immunized with NDV recombinant viruses rLa-NiV F, rLa-NiV G, or rLa-NiV F + rLa-NiV G [36]. Neutralization assays employing the recombinant pseudovirus rVSVΔG-eGFP-NiV_BD_ F/G revealed detectable NiV-specific antibodies as early as 2 weeks after the initial immunization. Antibody titers significantly increased after booster immunization (*p* < 0.01), peaking at 5–7 weeks post-vaccination (Figure 4B). Differences in immune responses were observed among the immunization strategies: the combined vaccination group (rLa-NiV F + rLa-NiV G) presented the highest neutralizing antibody levels, followed by the rLa-NiV G single vaccination group, whereas the rLa-NiV F single vaccination group presented the lowest antibody levels.

Further analysis suggested that the enhanced effect of the combined immunization may be attributed to the synergistic immune response between the F and G proteins, indicating that this combined approach is more effective in boosting the immune response. This study demonstrated that the recombinant virus rVSVΔG-eGFP-NiV_BD_ F/G is a reliable tool for neutralizing antibody detection and provides a safer and more efficient experimental platform for future immunological research.

### 3.5. Inactivated rVSVΔG-eGFP-NiV_BD_ F/G Induces Neutralizing Antibodies in Hamsters

**Figure 5:**
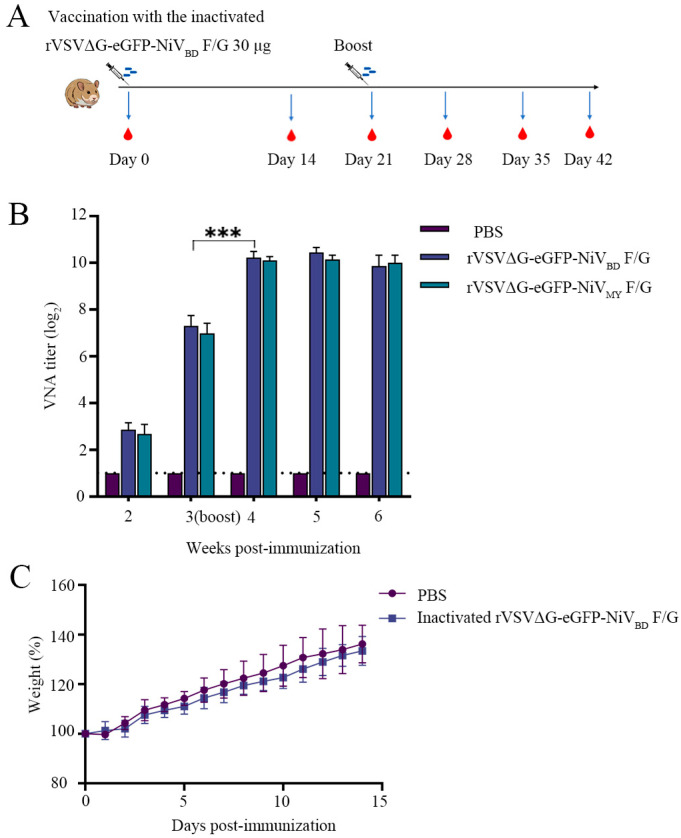
Vaccination schedule and neutralizing antibody response to the inactivated rVSVΔG-eGFP-NiVBD F/G: (**A**) Schematic representation of the vaccination schedule. The hamsters were immunized with 30 µg of the inactivated rVSVΔG-eGFP-NiV_BD_ F/G vaccine on day 0, followed by a booster dose at week 3. Serum samples were collected at weeks 2, 3, 4, 5, and 6 for subsequent analysis. (**B**) Viral-neutralizing antibody (VNA) titers (log2) in serum samples collected at various time points post-vaccination. VNA titers were measured at weeks 2, 3 (boost), 4, 5, and 6. The PBS group served as the control. The data are presented as the means ± SEMs. ***, *p* < 0.001. (**C**) Body weight changes (%) of hamsters following immunization with the inactivated rVSVΔG-eGFP-NiV_BD_ F/G or PBS control. Body weights were monitored daily for 14 days post-immunization.

Subsequently, we further evaluated whether the inactivated recombinant virus rVSVΔG-eGFP-NiV_BD_ F/G possesses immunogenicity. Serum samples were collected at weeks 2, 3, 4, 5, and 6 post-immunization, and humoral immune responses were assessed using neutralizing antibody assays (Figure 5A). The results showed that specific neutralizing antibodies against NiV were detectable in vaccinated animals as early as week 2 post-immunization, whereas no antibodies were detected in the control group (PBS) throughout the observation period. Following the booster dose, neutralizing antibody levels increased significantly and peaked two weeks later (week 5), reaching a titer of 1024, with a statistically significant difference (*p* < 0.0001) (Figure 5B).

To further evaluate the cross-neutralizing capacity of vaccine-induced antibodies, we employed the same construction strategy used for rVSVΔG-eGFP-NiV_BD_ F/G to generate and successfully rescue a recombinant virus expressing the F and G proteins of the Malaysian strain (NiV_MY_), rVSVΔG-eGFP-NiV_MY_ F/G, which was then used in neutralization assays. The results showed that sera from animals immunized with the inactivated rVSVΔG-eGFP-NiV_BD_ F/G vaccine exhibited potent neutralizing activity not only against the homologous Bangladeshi strain (NiV_BD_), but also against the heterologous Malaysian strain (NiV_MY_). Moreover, there was no significant difference in neutralizing efficacy between the two strains, indicating that the vaccine confers broad cross-protective potential. During the immunization period, safety monitoring of hamsters was conducted from day 1 to day 14 post-vaccination. The results showed that vaccinated hamsters exhibited no signs of clinical discomfort, such as significant changes in body weight (Figure 5C) or abnormal behavior. These findings indicate that the rVSVΔG-eGFP-NiV_BD_ F/G vaccine demonstrates good immunogenicity and safety in the golden Syrian hamster model, rapidly inducing high levels of specific neutralizing antibodies and showing promise as a candidate for preventing Nipah virus infection.

## 4. Discussion

Nipah virus (NiV) is a highly lethal zoonotic pathogen whose containment and study are severely constrained by the requirement for biosafety level 4 (BSL-4) laboratories, as well as the absence of approved vaccines or antiviral therapies. Its pathogenesis is primarily driven by the coordinated action of two surface glycoproteins: the attachment glycoprotein (G), which binds to ephrin-B2/B3 receptors on host cells, and the fusion glycoprotein (F), which mediates membrane fusion. This coordinated interaction is essential for viral entry, and both glycoproteins serve as major targets for neutralizing antibodies, making their immunogenic properties critical determinants of vaccine efficacy. To bypass the limitations of BSL-4 containment and to enable mechanistic studies of NiV entry, we engineered a replication-competent chimeric virus, rVSVΔG-eGFP-NiV_BD_ F/G, using reverse genetics.

Functional characterization demonstrated that the recombinant virus preserves key biological properties of NiV, including receptor-specific cell tropism and the ability to induce syncytium formation, thereby closely resembling aspects of natural infection. Although its replication is attenuated relative to wild-type VSV—likely due to the absence of the native VSV G protein, which is critical for efficient intercellular viral spread [20]—rVSVΔG-eGFP-NiV_BD_ F/G effectively recapitulates the receptor engagement and membrane fusion stages of the NiV life cycle. This makes it a valuable tool for studying NiV entry and immune recognition under enhanced biosafety but more accessible conditions, offering a practical alternative to live NiV work in BSL-4 laboratories and supporting a wider range of research applications.

Neutralization assays using NiV-specific monoclonal antibodies (HENV-26/32) confirmed the specific recognition of the recombinant virus F/G proteins, validating their potential for antibody screening applications. Importantly, although this recombinant virus lacks other NiV structural proteins and relies on the VSV backbone for replication, our data indicate that the surface-expressed NiV F and G glycoproteins retain a native-like conformation. This is supported by the strong and specific neutralizing responses observed with the monoclonal antibodies HENV-26 and HENV-32, which are known to target conformational epitopes on natural NiV glycoproteins. The robust neutralization activity strongly suggests that the recombinant glycoproteins maintain key structural features essential for receptor binding and immune recognition. We used a eukaryotic virus expression system, which, unlike other expression systems (such as bacterial or insect systems), could produce glycoproteins that closely mimic their native conformation during NiV infection in host cells.

To comprehensively characterize the biological properties and host adaptability of the recombinant virus, we first performed infectivity assays across a diverse panel of cell lines derived from various animal species to assess its tissue and species tropism. The results demonstrated that rVSVΔG-eGFP-NiV_BD_ F/G effectively infected multiple mammalian cell lines—including BHK, Vero, HEK293, CRFK, EqEK, MDBK, and PAM—indicating a broad host range and potential for cross-species transmission. This finding aligns with NiV’s known ecological feature of infecting diverse natural hosts. Conversely, no significant infection was observed in PK-15, MDCK, CHO, and ST cells, highlighting the virus’s tissue-specific tropism and providing a basis for identifying possible intermediate hosts.

Building upon these results, we further investigated the biological characteristics of the recombinant virus and its receptor usage, and we conducted receptor tropism studies. Specifically, we systematically evaluated the susceptibility of MDCK cells expressing various simian (A1, A5, B1, B2, B3) and human (B1, B2) EFN receptors to infection by the recombinant virus. In the analysis of the susceptibility of cells expressing EFN receptors from different sources, we observed that MDCK cells transfected with sEFN B1, sEFN B2, sEFN B3, huEFN B1, or huEFN B2 exhibited varying degrees of susceptibility to the recombinant virus rVSVΔG-eGFP-NiV_BD_ F/G, as indicated by the expression of eGFP fluorescence signals. Notably, cells expressing sEFN B2, sEFN B3, and huEFN B2 showed high levels of eGFP expression along with prominent syncytium formation as previously reported [37,38]. In contrast, cells expressing sEFN B1 or huEFN B1 were also susceptible to infection but displayed relatively lower levels of eGFP expression and less pronounced cell fusion. This observation is also consistent with the results obtained by the Benhur Lee group using luciferase-based assays [39]. These findings suggest that although EFNB1 may play a role in NiV entry, its efficiency is significantly lower than that of EFNB2 and EFNB3. Meanwhile, cells expressing sEFN A1 or A5 showed no signs of infection or morphological changes, further confirming EFNB2 and EFNB3 as the primary receptors critical for viral entry, whereas EFNB1 may possess a conditional, auxiliary role in mediating viral entry. Notably, previous studies based primarily on bright-field microscopy may have underestimated the functionality of EFNB1 due to limited detection sensitivity. In contrast, the eGFP-labeled recombinant virus system employed in this study offers significantly enhanced detection sensitivity, enabling the identification of low-efficiency membrane fusion events that are difficult to detect using conventional methods, thereby allowing for a more accurate evaluation of low-level infections. Taken together, our findings not only deepen the understanding of the potential role of EFNB1 in NiV infection but also underscore the unique advantages of fluorescence labeling systems in receptor tropism research, providing a novel technical approach and theoretical foundation for elucidating the receptor usage profile of NiV.

Overall, the cell and tissue tropism of Nipah virus (NiV) is primarily determined by its entry receptor. However, interactions between the virus and host proteins during replication and release also influence its tropism in tissues. Compared to NiV, the chimeric virus exhibits differences in its role during various stages of the viral lifecycle. Specifically, the chimeric virus can only mimic NiV’s behavior during the entry phase, while other stages, such as replication and release, still rely on the VSV system. Therefore, our chimeric virus is primarily capable of effectively simulating NiV’s entry process, providing an important experimental model for further studying NiV’s cell tropism and entry mechanisms.

Previous studies on recombinant VSV viral vector vaccines have mainly used a strategy of expressing a single glycoprotein (NiV F or G). For example, the VSV-NiV G vaccine developed by Geisbert’s team demonstrated good immune protection efficacy in non-human primate models [28]. In addition, Spiropoulou and others demonstrated that a single-dose vaccine expressing either NiV G or NiV F in a replication-defective VSV vector fully protected hamsters from lethal NiV challenge [27]. However, such single-glycoprotein vaccines have two major limitations: first, most attenuated recombinant viruses require the VSV G protein for amplification, which inevitably leads to contamination with residual VSV G in the harvested viral preparations; second, the vaccine yield is often unstable, severely constraining large-scale production and practical application.

**Figure A2:**
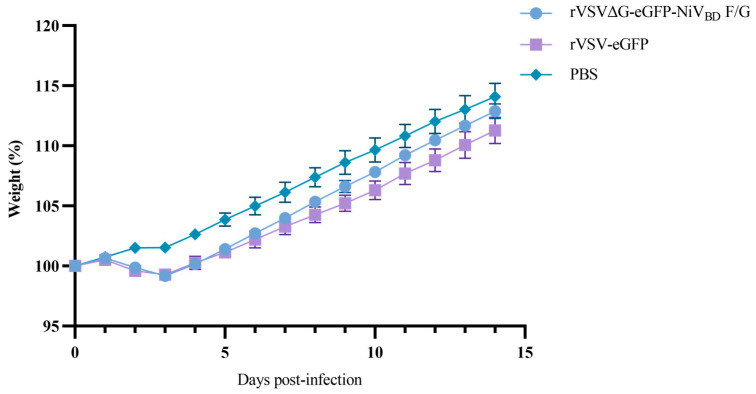
Body weight changes (%) of mice following intramuscular immunization with rVSVΔG-eGFP-NiV_BD_ F/G, rVSV-eGFP, or PBS control. Body weights were monitored daily for 14 days post-infection.

It is worth noting that although the recombinant virus constructed in this study, rVSVΔG-eGFP-NiV_BD_ F/G, does not contain the VSV G gene, it retains a degree of replication capacity. Since viral entry mediated by the NiV F and G proteins depends on ephrinB2 and ephrinB3 receptors—both of which are highly expressed in the central nervous system—this recombinant virus may still pose a potential risk of neuroinvasion, which cannot be overlooked. To further evaluate the in vivo safety of rVSVΔG-eGFP-NiV_BD_ F/G, intramuscular injection, a conventional immunization route, was employed in both mice and hamsters. The results showed that mice exhibited no obvious clinical symptoms or mortality, although their body weight slightly decreased compared with the PBS control group after intramuscular injection (Figure A2). Meanwhile, a certain degree of pathogenicity was observed in hamsters, which was correlated with their natural susceptibility to NiV. Based on these findings, we believe that live-virus vaccines still carry potential safety risks. Therefore, the virus was further inactivated in this study to eliminate safety concerns associated with replication-competent vectors, and the immunogenicity of the inactivated virus was subsequently evaluated. Animal experiments demonstrated that a prime-boost immunization regimen significantly increased neutralizing antibody titers, reaching as high as 1024 (*p* < 0.001), indicating that the recombinant virus retained strong immunogenicity even in its inactivated form.

In conclusion, the successful construction of the rVSVΔG-eGFP-NiV_BD_ F/G recombinant virus platform not only provides a reliable experimental tool for research on Nipah virus and other henipaviruses but also offers important technical support for vaccine development and studies on viral infection mechanisms. This platform will become a key tool in pandemic prevention and antiviral research, providing effective support for the development of vaccines against Nipah virus and other henipaviruses.

## 5. Conclusions

The establishment of the rVSVΔG-eGFP-NiV_BD_ F/G platform represents an advancement in Nipah virus (NiV) research. Its dual advantages of sensitivity and functional authenticity not only accelerate the development of neutralizing antibodies and vaccines but also provide a versatile tool for investigating shared invasion mechanisms within the Henipavirus genus. By elucidating the relationship between receptor specificity variations and host adaptability, this study offers new insights into predicting the cross-species transmission risks of emerging zoonotic viruses. Overall, this platform might provide a robust foundation for future research and control strategies targeting the Nipah virus and other henipaviruses.

## Data Availability

The data that support the findings of this study are openly available in this manuscript.
